# Plasma oxytocin explains individual differences in neural substrates of social perception

**DOI:** 10.3389/fnhum.2015.00132

**Published:** 2015-03-17

**Authors:** Katie Lancaster, C. Sue Carter, Hossein Pournajafi-Nazarloo, Themistoclis Karaoli, Travis S. Lillard, Allison Jack, John M. Davis, James P. Morris, Jessica J. Connelly

**Affiliations:** ^1^Department of Psychology, University of VirginiaCharlottesville, VA, USA; ^2^Kinsey Institute and Department of Biology, Indiana UniversityBloomington, IN, USA; ^3^Robert M. Berne Cardiovascular Research Center, University of VirginiaCharlottesville, VA, USA; ^4^Yale Child Study Center, Yale UniversityNew Haven, CT, USA; ^5^Department of Psychiatry, University of Illinois at ChicagoChicago, IL, USA

**Keywords:** oxytocin, social cognition, fMRI, neuroendocrinology, neuroimaging, individual differences

## Abstract

The neuropeptide oxytocin plays a critical role in social cognition and behavior. A number of studies using intranasal administration have demonstrated that oxytocin improves social perception. However, little is known about the relationship between individual differences in endogenous levels of oxytocin and social cognition. In the current study, we assessed the relationship between endogenous oxytocin and brain activity during an animacy perception paradigm. Thirty-seven male participants underwent scanning and provided a blood sample for oxytocin analysis. In line with previous research, perception of animacy was associated with activations in superior temporal sulcus, inferior frontal gyrus, and medial prefrontal cortex (mPFC). Notably, participants’ levels of plasma oxytocin robustly predicted activation in areas critical for social cognitive processes, such that higher oxytocin levels were related to increased activity in dorsal mPFC, ventral mPFC, dorsolateral PFC, superior temporal gyrus, and temporoparietal junction (TPJ), suggesting differential processing of social stimuli. Together these results show that stable variations in endogenous oxytocin levels explain individual differences in social perception.

## Introduction

There is growing interest in understanding social cognition from the level of individual biology. Differences in an individual’s ability to perceive, interpret, and act upon social information is well-established in populations whose social deficits are a hallmark of their disorder (e.g., autism, schizophrenia). Interest in establishing the biological roots of these disorders have resulted in the use of endogenous, molecular predictors (e.g., genes, hormones) to explain variations in behavior and provide a mechanistic explanation for these social deficits. It is unclear, however, how the range of social cognitive variability in healthy populations might be explained by these same biologically-based predictors. The current study attempts to validate a biomarker that predicts individual differences in social cognition in healthy (neurotypical) participants.

One such candidate biomarker is oxytocin (OT), a neuropeptide important for pair bonding and prosocial behaviors. Recent attention has been devoted to intranasal administration of OT and its effects on social cognition. Intranasal OT promotes prosociality by increasing trust (Kosfeld et al., 2005), generosity/reciprocity (Zak et al., 2007), and one’s ability to infer mental states from nonverbal cues (Domes et al., 2007; Guastella et al., 2010). Moreover, OT administration also improves social perceptual processing such as enhancing recognition of facial expressions (Marsh et al., 2010) and increasing sensitivity to biological motion (Kéri and Benedek, 2009).

While the link between OT administration and social cognition is becoming clear, the role of *endogenous* OT has been largely ignored. An increasing number of double-blind, random control trials are being conducted to assess how behavior and cognition are modulated by exogenous OT administration (MacDonald et al., 2011), but fewer studies leverage individual variability in plasma OT levels to understand how behavior and cognition are affected in the natural environment. Considerable interindividual variability in endogenous OT levels exists: plasma OT levels are heritable (Rubin et al., 2014) and show remarkable stability over time. Feldman and colleagues (Feldman et al., 2007) assessed plasma OT levels in individuals at multiple time points over 6–9 months and obtained within-subject correlations of *r* = 0.92–*r* = 0.96. On shorter time scales, OT levels are also sensitive to contexts and have been shown to be modulated by social interactions (Feldman et al., 2010).

Importantly, endogenous levels of OT are also predictive of social behavior. Of the endogenous OT studies that exist, a number have shown positive relationships between plasma OT and affiliative parenting styles (Gordon et al., 2010), prosocial interactions with one’s romantic partner (Gouin et al., 2010), trust (Zhong et al., 2012), and secure attachment styles (Tops et al., 2007). A key question, however, is whether plasma OT levels are similarly predictive of basic social cognitive functioning. Low plasma levels have been observed in clinical populations with marked social deficits, such as in autism (Modahl et al., 1998), and greater OT levels have been associated with more positive social outcomes, such as in schizophrenia (Rubin et al., 2010). Recent work suggests that this relationship may transcend clinical borders (Parker et al., 2014) and that endogenous OT levels support the socio-emotional and perceptive abilities essential for social cognition (Atzil et al., 2012). We aim to directly test this association in a sample of healthy adults.

The current study uses functional neuroimaging to probe subtle variability in social cognition that might not be captured in overt behavior. In a neurotypical population, performance on social cognitive tasks is often at ceiling. Many of the promotional effects of OT administration on social cognition have been observed only when proficiency on the task at hand is low, either due to autism-related deficits (Guastella et al., 2010) or to individual differences in social proficiency (Bartz et al., 2010). A recent report by Parker and colleagues observed that plasma OT is associated with social function in young children regardless of disease or risk status for disease (Parker et al., 2014); however, the associations between OT and behavior in the control group were modest and the significant relationship was likely driven by the added variability in social function provided by sampling from both healthy children and those with autism. Given the restricted range in performance in healthy populations, traditional measures are less suitable for drawing associations with biological predictors. In comparison, variations in neural activity have greater sensitivity and can be observed in the absence of behavioral differences; for instance, Kaiser et al. (2010) demonstrated similar neural signatures for people with autism and their unaffected siblings. While both groups shared a genetic susceptibility to autism observable at the neural level, this susceptibility was not adequately captured using behavioral measures. Moreover, functional neuroimaging has also proven useful in understanding the impact of intranasal OT administration on social cognition (see Bethlehem et al., 2013 for a comprehensive review).

In this study we used fMRI in concert with a task where participants view films of geometric shapes interacting in an animate fashion, a variation on Heider and Simmel’s classic paradigm (Heider and Simmel, 1944). Because participants spontaneously attribute mental states to these shapes, this task reliably elicits activations in a network of brain regions important for social cognition and perception, including the medial prefrontal cortex (mPFC), superior temporal sulcus, temporoparietal junction (TPJ), temporal poles, and the fusiform gyrus (FG; Castelli et al., 2000, 2002; Schultz et al., 2003). Performance on this task has been shown to be affected in people with autism, with children with autism providing fewer mental state attributions (Abell et al., 2000), and adults with autism showing reduced brain activity in regions described above (Castelli et al., 2002). This pattern of deficits allows us to make predictions about variability in healthy populations. Based on our prediction that plasma OT levels could serve as a valid biomarker of social cognitive functioning in healthy individuals, we hypothesized that higher plasma OT levels would predict greater brain activity in this task, specifically in regions supporting social cognition.

## Materials and Methods

### Participants

Forty males (aged 18–29) participated in this experiment prior to September 2013 as part of a larger imaging genetics study. Participants were majority Caucasian, with 2 African American, 2 East Asian participants, and 2 biracial participants (Black/White, Hispanic/White). Participants also completed a blood draw and a set of questionnaires for the larger study, and were paid $50. Informed consent was obtained from all participants according to the guidelines set by the Health Sciences Institutional Review Board at the University of Virginia. Three participants’ data were excluded^1^ from analyses (all Caucasian) due to high (>2–4 SD) OT levels, leaving 37 participants for the main analysis (mean age = 23.69).

### Plasma Oxytocin

To minimize temporal variability, 8 ml of blood was collected from participants between 10:00–11:00 am in a BD*^TM^* P100 blood collection tube (BD Diagnostics, Franklin Lakes, NJ). These tubes contain proprietary stabilizers that solubilize immediately upon blood collection which prevents peptide degradation during collection and at other downstream timepoints. Samples were centrifuged within 1 h of collection per the manufacturer’s protocol. The plasma was aliquoted by milliliter into cryotubes, frozen overnight at −20°C and then stored indefinitely at −80°C. In 2011, one tube from the first 28 samples was sent to the laboratory of Sue Carter, then at the University of Illinois in Chicago for analysis; in 2013 the same 28 samples and an additional 12 samples were sent to the laboratory of Sue Carter, now at the University of North Carolina.

OT levels were measured using a commercially available enzyme immunoassay (EIA: Enzo Life Sciences Inc., Farmingdale, NY), which is sensitive (minimal detection levels > 12 pg/ml OT) and specific with cross-reactivity between OT and AVP < 0.04% (Rubin et al., 2014). Direct measurement of OT was performed following the manufacturer’s protocols and samples were diluted 1:2 as described previously (Kramer et al., 2004). Samples were not extracted prior to analysis, as extraction has been shown to precipitate the majority of the OT in the blood (Martin and Carter, 2013). Samples were assayed blind to subject information and all samples used in analysis were run at the same time. OT plasma levels ranged from 124.1 to 1483.6 pg/ml, with a mean of 365.35 (SD = 248.97). After removing the three outliers (range: 895.5–1483.6 pg/ml), the new mean was 307.28 (SD = 122.61). The inter- and intra-assay coefficients of variability were 1.19% and 14.54%, respectively. Samples that were run 2 years apart in two different lab settings correlated at Pearson’s *r* = 0.98 indicating remarkable stability of the samples and reliability of the assay.

Validation of this EIA procedure has been described in (Carter et al., 2007) and repeated in several laboratories in studies in plasma from humans (e.g., Feldman et al., 2007, 2010; Gordon et al., 2010; Gouin et al., 2010; Rubin et al., 2014), nonhuman primates, rats and voles (Kramer et al., 2004). Tests for parallelism have been conducted using serial dilutions of plasma. Accuracy has been assessed by spiking samples with standards of known concentrations of oxytocin. Specificity of the antibody in the Assay Designs EIA kit was previous validated by our group using high pressure liquid chromatography (for data and details, see Carter et al., 2007).

### Social Cognition Task

Video stimuli were adapted from (Schultz et al., 2003) and included 15 s clips of geometric shapes interacting in a goal-oriented (Animate condition) or random fashion (Random condition). Each condition consisted of 8 clips (16 total), interleaved with a jittered intertrial interval of approximately 1 s. In total, each run lasted 3 min and 56 s. Participants were asked to actively attend to the video clips, but were not given any additional instructions. Verbal confirmation immediately pre- and post-run ensured participants were actively attending to the task. Online monitoring of eye movements between scan volumes served as another confirmation of attentiveness.

### Imaging Acquisition and Analysis

Participants were scanned using a Siemens Magnetom Trio 3T MRI scanner and 12-channel head coil. A t1-weighted gradient-echo structural image (MPRAGE ; TR = 1900 ms; TE = 2/53; FOV = 250 mm; resolution = 1 × 1 × 1 mm) was acquired at the beginning of the scan, followed by the T2*-weighted echo-planar (EPI) functional images (TR = 2000 ms; TE = 40 ms; resolution = 3 × 3 × 4.2 mm; flip angle = 90 degrees; 28 slices), coplanar with the structural image. A total of 128 functional brain volumes were acquired.

Imaging analysis was conducted using GLM models in FSL v5.0 (FMRIB software).^2^ Preprocessing steps included motion-correction, brain extraction, coregistration to each participant’s structural scan, spatial smoothing with a Gaussian kernel of 5 mm FWHM, grand-mean intensity normalization, and normalization into standard (Montreal Neurological Institute (MNI)) space. Each subject’s first level linear model was created using 15 s timecourses for each of the Animate and Random conditions convolved with a standard gamma HRF function. For the contrast of Animate-Random, a contrast of parameter estimates (COPE) was computed for all subjects. These individual COPEs were then used in the higher level group models.

Two mixed-effects group models were constructed. First, a main effect of group activation for the Animate-Random contrast was computed to identify brain regions that were reliably activated by the task. Additionally, we investigated the role of plasma OT as a predictor of neural activity in the Animate-Random contrast; OT levels were demeaned and added to the GLM as a predictor. A whole-brain analysis was conducted. *Z* (Gaussianised *T*) statistic images were thresholded using clusters determined by *Z* > 2.3 and a corrected cluster significance threshold of *p* < 0.05 (Worsley, 2001). Because our analysis with OT is novel and somewhat exploratory, we did not conduct an a priori region-of-interest analysis, but instead relied solely on a more conservative whole-brain analysis.

## Results

**Figure 1:**
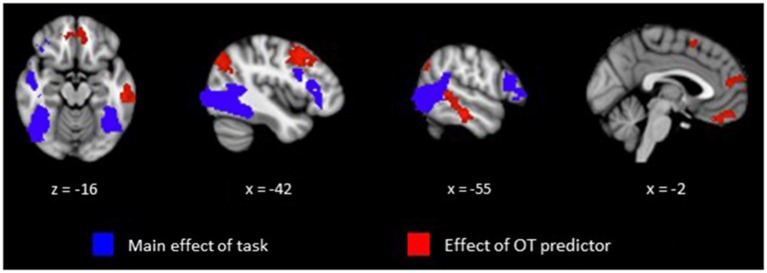
**Clusters representing positive activations from the Animate vs. Random contrast from whole-brain analysis with cluster thresholding**. Activations in blue represent positive task-related activity for Animate relative to Random motion trials. Activations in red represent regions that show a significant relationship between level of plasma OT and positive task-related activity for Animate relative to Random motion trials.

Our primary analysis focused on whether OT can predict activation differences in brain regions sensitive to perception of animate relative to random motion. Plasma OT was entered into our model as a predictor and task-related activations were computed with and without OT as predictor. Figure 1 (blue clusters) and Table 1, panel A show clusters of activity derived from the initial model of mean group activation for the Animate-Random contrast. Consistent with prior research (Castelli et al., 2000; Schultz et al., 2003), the task elicited activity in lateral occipital regions extending into the superior temporal gyrus and ventrally into the fusiform. Also active were lateral frontal regions, including inferior frontal gyrus extending into the frontal poles.

**Table 1:** **Regions demonstrating greater BOLD response to Animate vs. Random displays**.

Contrast	Region	Hemisphere	*x*	*y*	*z*	*Z*	*k*
**A. Group mean**
	STS/lateral occipital/fusiform	R	30	−98	−6	7.77	8926
		L	−32	−94	−6	7.71	4481
	IFG/frontal poles	R	48	24	16	5.85	3621
		L	−50	24	16	4.62	1099
	SPL/Angular gyrus	R	30	−50	38	3.53	322
**B. OT Predictor**
	dlPFC	L	−40	18	46	4.60	1114
	M/STS	L	−56	−28	−18	4.51	769
	dmPFC	medial	−14	64	22	4.61	623
	TPJ/angular gyrus	L	−46	−72	42	4.14	491
	vmPFC	medial	−6	52	−20	3.74	344

*Legend: Table 1 reports the locations of peak voxels for significant clusters, p < 0.05, corrected for multiple comparisons. Coordinates are presented in MNI space; k = number of voxels. Panel A represents regions that are significantly activated for the Animate-Random contrast. Panel B represents regions within the Animate-Random contrast that are significantly predicted by modeling OT levels as a continuous predictor. STS = superior temporal sulcus; IFG = inferior frontal gyrus; SPL = superior parietal lobule; dlPFC = dorsolateral prefrontal cortex; M/STS = middle/superior temporal sulcus; dmPFC = dorsomedial prefrontal cortex; TPJ = temporoparietal junction; vmPFC = ventromedial prefrontal cortex*.

**Figure 2:**
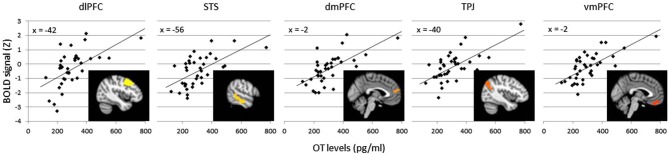
**Average BOLD signal values (Z scores) in the Animate-Random contrast for clusters significantly predicted by OT (clusters from Table 1B), plotted against OT levels to illustrate a positive association**. Variation in cluster color represents independent clusters.

The effects of plasma OT as a predictor of neural activity for the Animate-Random contrast are illustrated in Figure 1 (red clusters) and described in Table 1, panel B. After modeling OT as a predictor in our whole-brain analysis, five large (all >300 voxels) significant clusters were identified, located in regions previously identified to be critical to social cognitive processes: dorsal medial prefrontal cortex (dmPFC), ventromedial prefrontal cortex (vmPFC), left TPJ/angular gyrus extending into the left lateral cortex, left dorsolateral prefrontal cortex (dlPFC) extending medially into the paracingulate, and left middle/superior temporal gyrus (STS). To demonstrate the direction of the relationship between OT and brain activity, we registered these significant clusters back to participants’ native space, extracted values for the Animate-Random contrast and plotted them against OT levels. As seen in Figure 2, these relationships are positive, indicating that participants whose OT levels were higher had greater brain activity for Animate displays than for Random displays.

## Discussion

As predicted, the current results revealed that plasma OT is associated with greater activity in brain regions critical to social cognition such as mentalizing, experience-sharing, and empathy (Zaki and Ochsner, 2012). Previous work by Tavares and colleagues (Tavares et al., 2008) has shown that neural activity within a similar task is modulated via selective attention, such that directing one’s attention toward the social aspects of this task (i.e., mental state attributions) engages the “social brain” to a greater extent than directing attention toward its physical aspects (i.e., the spatial properties of the stimuli). While we did not manipulate attention in our task, the brain activity in regions predicted by OT levels parallels results from the condition in Tavares et al. (2008) in which attention was directed to social aspects of the stimuli (i.e., greater activation in TPJ, STS, dmPFC). This suggests that participants with higher endogenous OT levels are naturally attending to the more social aspects of the task. Broadly, we believe these results demonstrate that natural variations in OT levels may predict an individual’s sensitivity to social information.

These results have several implications for the existing OT administration literature. For one, our findings may help us to interpret some of the prior conflicting findings arising from intranasal OT administration. While plasma levels of OT have largely been ignored in administration studies, it has been previously speculated that exogenous OT administration may interact with endogenous levels to influence behavior (Bartz et al., 2011). Prior work has shown that OT effects may be dose-dependent (Cardoso et al., 2013), and non-linear (Zhong et al., 2012). Our own sample of healthy, young, male subjects showed considerable variability in plasma OT levels—so much so that we excluded 3 outliers from our relatively small sample. It is not clear that exogenous administration will similarly impact individuals when such high variability in endogenous levels of OT exists. Our finding that these levels have functional significance suggests that OT plasma levels are tonic individual differences that should be measured and considered in relation to pharmacological manipulations.

Our study comes with several caveats. As with any proof-of-concept study, a number of questions remain unanswered. First, like many other OT studies, our sample consists solely of male subjects. This is problematic since sex differences in the effects of OT administration on social cognition have been observed (Domes et al., 2010), potentially due to interactions with female sex steroids (McCarthy and Pfaus, 1996). Further, plasma OT levels may vary as a function of the menstrual cycle, with luteal phase decreases that are not observed in women taking oral contraceptives (Salonia et al., 2005). For these reasons, OT studies involving women participants should be optimized to control for variations in menstrual cycle status and conceptive use. In the context of the current work, it is unclear whether we would expect sex-differences in our simple, non-valenced task; an OT administration study with a similar task (perception of biological motion) did not find sex differences (Kéri and Benedek, 2009), nor are sex differences usually reported more broadly for this task. Thus, we have no direct predictions about sex effects and hope these will be empirically investigated in the future, despite the difficulties in properly assessing plasma OT in women.

Another issue concerns the relationship between peripheral (plasma) OT and central OT. Because endogenous OT cannot cross the blood-brain barrier, it is unclear how well plasma levels are associated with levels in the CNS; peripheral levels are regulated by the posterior pituitary, while central levels are regulated by nuclei within the hypothalamus (Neumann, 2008). The evidence on their association is mixed, with some animal models showing little association between plasma OT and OT levels in CSF (Winslow et al., 2003), while others show coordinated release of OT both peripherally and in the supraoptic nucleus of the hypothalamus (Landgraf and Neumann, 2004). However, questions of mechanism are not limited to plasma OT studies; research involving intranasally administered OT has similar mechanistic gaps. In fact, it had not been shown until recently that intranasally administered OT is taken up by the brain (Neumann et al., 2013). Moreover, the particular route through which intranasally administered OT reaches the brain has not been conclusively identified (Evans et al., 2014). The association between OT—whether observed or manipulated—and behavior is ultimately the most important piece of evidence for its neuromodulatory role.

In conclusion, we present initial evidence that plasma OT levels are significant predictors of individual differences in neural response to social stimuli. While the importance of OT for social cognition and behavior has been demonstrated in a number of exogenous administration studies, presumably these effects are meaningful only insofar that they can be shown to occur within the naturally-occurring variability in endogenous levels of OT. We are the first to show this at the neural level. Specifically, we show that plasma OT levels are strongly, positively associated with activity in the brain’s centers for social cognitive processing. Given that endogenous OT levels vary substantially between people and are stable over time, plasma OT could serve as a biological indicator of sensitivity to social information. Further study of endogenous OT may provide insight into the biological mechanisms underlying individual differences in this domain.

## Author Contributions

SCC, JMD, JPM and JJC developed the study concept. All authors contributed to the study design. KL, AJ, JPM and JJC wrote the article. Data collection and analysis were performed by AJ, KL, TK, TSL and HP-N. All authors are responsible for the content of this article.

## Conflict of Interest Statement

The authors declare that the research was conducted in the absence of any commercial or financial relationships that could be construed as a potential conflict of interest.

## Footnotes

^1^An alternate treatment of extreme OT values is log-transformation. We include a summary of these results in the Supplementary Data section.

^2^www.fmrib.ox.ac.uk/fsl
